# Development and internal validation of a nomogram to predict temporary acute agitated delirium after surgery for chronic subdural hematoma in elderly patients: an analysis of the clinical database

**DOI:** 10.3389/fneur.2024.1394476

**Published:** 2024-05-07

**Authors:** Peng Wang, Shasha Yang, Jianqiao Zheng, Jinjiang Lu, Nan Li, Jing Zhang

**Affiliations:** ^1^Department of Neurosurgery, Yuebei People’s Hospital of Shantou University Medical College, Shaoguan, China; ^2^Department of Pathology, Yuebei People’s Hospital of Shantou University Medical College, Shaoguan, China; ^3^Doctor of Medicine, Department of Emergency Medicine, General Hospital of Northern Theater Command, Shenyang, China; ^4^Intensive Care Unit, Yuebei People’s Hospital of Shantou University Medical College, Shaoguan, China

**Keywords:** CSDH, delirium, nomogram, clinical impact curve, DCA

## Abstract

**Background:**

This study aimed to develop a nomogram for predicting temporary acute agitated delirium after surgery in patients with chronic subdural hematoma (CSH) without neurological compromise and hospitalized in the neurosurgery.

**Methods:**

We included 289 patients with chronic subdural hematoma (CSH) from the medical information system of Yuebei People’s Hospital of Shaoguan City, Guangdong Province, and collected 16 clinical indicators within 24 h of admission. We used the least absolute shrinkage and selection operator (LASSO) regression to identify risk factors. We established a multivariate logistic regression model and constructed a nomogram. We performed internal validation by 1,000 bootstrap samples; we plotted a receiver operating curve (ROC) and calculated the area under the curve (AUC), sensitivity, and specificity. We also evaluated the calibration of our model by the calibration curve and the Hosmer–Lemeshow goodness-of-fit test (HL test). We performed a decision curve analysis (DCA) and a clinical impact curve (CIC) to assess the net clinical benefit of our model.

**Results:**

The nomogram included alcoholism history, hepatic insufficiency, verbal rating scale for postoperative pain (VRS), pre-hospital modified Rankin Scale (mRS), and preoperative hematoma thickness as predictors. Our model showed satisfactory diagnostic performance with an AUC value of 0.8474 in the validation set. The calibration curve and the HL test showed good agreement between predicted and observed outcomes (*p* = 0.9288). The DCA and CIC showed that our model had a high predictive ability for the occurrence of postoperative delirium in patients with CSDH.

**Conclusion:**

We identified alcoholism, liver dysfunction, pre-hospital mRS, preoperative hematoma thickness, and postoperative VRS pain as predictors of postoperative delirium in chronic subdural hematoma patients. We developed and validated a multivariate logistic regression model and a nomogram.

## 1 Introduction

Chronic subdural hematoma (CSDH) is a chronic space-occupying lesion in the brain, formed by the accumulation of blood between the arachnoid and the dura mater, usually occurring in the third week after traumatic brain injury (1). It is common in elderly people (2), and its incidence has been increasing year by year in recent years (3). The incidence of CSDH in the general population is 1.7/100,000 to 20.6/100,000 per year (3–5). There are several surgical methods to remove CSDH, mainly including twist drill craniostomy (TC), burr hole craniostomy (single or double) (BC), and craniotomy. A novel treatment for symptomatic chronic CSDH is endovascular embolization of the middle meningeal artery (MMA) (6). Burr hole closed drainage is the most widely used surgical method for CSDH (7). Studies have shown that postoperative delirium (POD) is one of the common complications after this disease, clinically manifested as sleep disturbance, anxiety, attention deficit or decline, etc., often occurring on postoperative 1–3 days, which can lead to prolonged hospital stay, increased medical costs, and even affect the mortality of patients (8–10). Establishing a prediction model for postoperative delirium of chronic subdural hematoma will help to screen high-risk populations, provide timely intervention measures, and may help to reduce the incidence, severity, and duration of delirium. Therefore, the application of delirium prediction model is of great significance for preventing the adverse outcomes caused by delirium, and has an important impact on the treatment and rehabilitation of elderly patients with chronic subdural hematoma. However, there are few studies on the risk nomogram prediction model for postoperative delirium in elderly patients with chronic subdural hematoma (11). This study adopts a retrospective research design, and collects relevant data to determine the occurrence and risk factors of delirium in elderly patients with chronic subdural hematoma.

## 2 Materials and methods

### 2.1 Subjects

**Figure 1:**
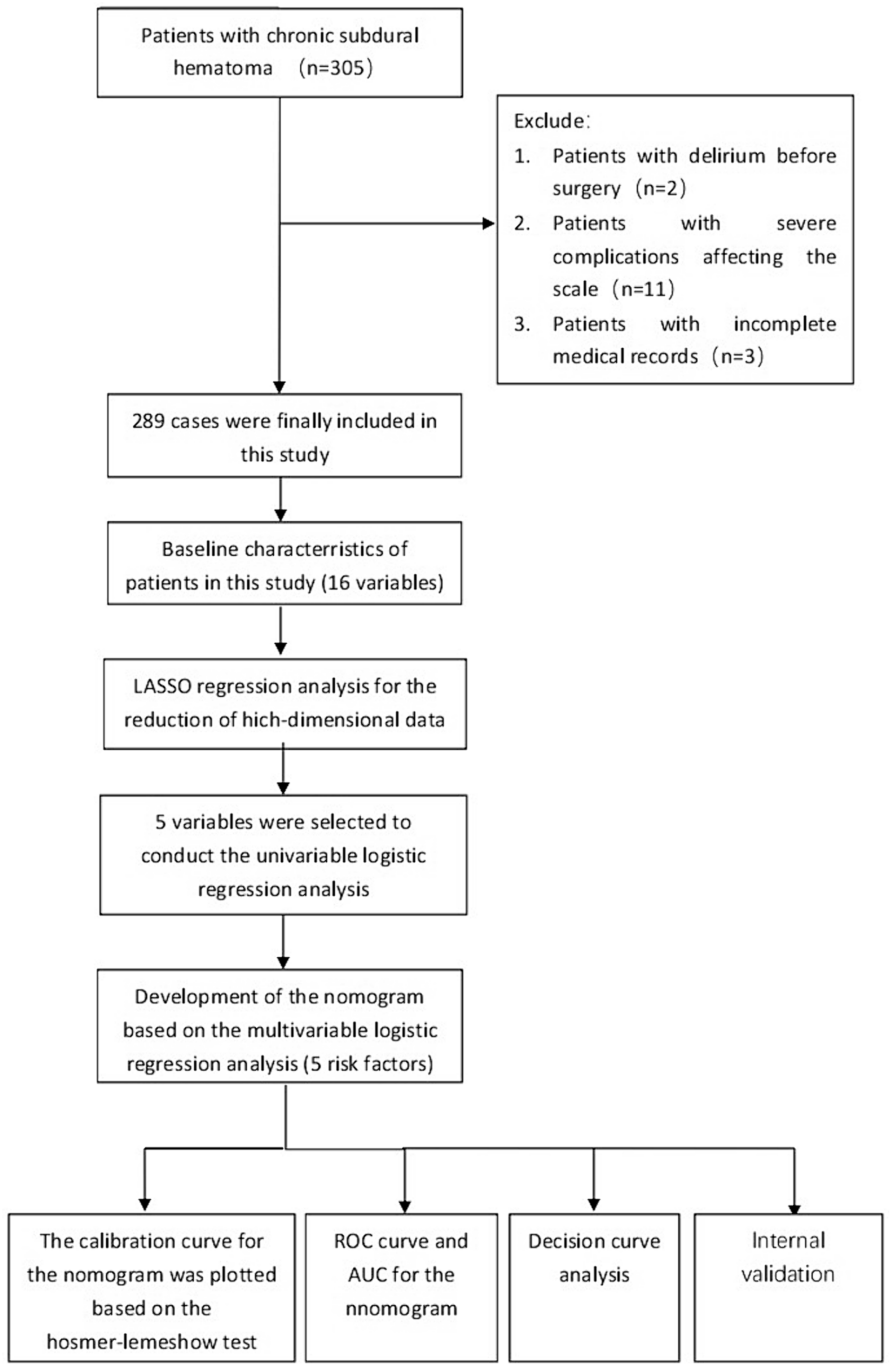
The flowchart for selection procedure of patients with CSDH who underwent surgical treatment.

A retrospective study design was adopted, and 305 patients with chronic subdural hematoma admitted to the Yuebei People’s Hospital from January 2017 to December 2022 were selected as the study subjects. Inclusion criteria: (1) diagnosed as chronic subdural hematoma by preoperative brain CT or MRI examination, and confirmed as chronic subdural hematoma after surgery; (2) underwent chronic subdural hematoma burr hole drainage under general anesthesia; (3) able to communicate normally. Exclusion criteria: (1) preoperative Mini-Mental State Examination (MMSE) score <10 points (2 cases); (2) in a coma or deep sedation state before or after surgery, Richmond Agitation-Sedation Scale (RASS) score <−3 points (11 cases); (3) important clinical information incomplete (3 cases). This study complied with the requirements of the Declaration of Helsinki. Finally, 289 patients were included in this study (Figure 1).

### 2.2 Methods

#### 2.2.1 Research methods

The enrolled patients were assessed within 8 h after surgery, and the diagnostic criteria were based on the 5th edition of the Diagnostic and Statistical Manual of Mental Disorders (DSM-5) by the American Psychiatric Association. The diagnosis of postoperative delirium was made by a joint consultation of psychiatrists and neurosurgeons. Previous studies have shown that advanced age, personal history (smoking, alcohol abuse), past medical history (liver dysfunction, hypertension, diabetes, heart disease, etc.), mRS score, and severity of cerebral hemorrhage may be closely related to the occurrence of postoperative delirium (12, 13). Based on this, the clinical data of the patients were collected, as follows: (1) general data: age, gender, smoking history, alcohol abuse history, liver dysfunction, and stroke. The diagnoses of alcoholism, liver dysfunction, and previous stroke were made by the medical professionals at Yuebei People’s Hospital, including psychiatrists, gastroenterologists, and neurologists. (2) Clinical disease and surgery-related data: including operation time, postoperative VRS rating, and restraint belt restraint. (3) Severity of subdural hematoma data: including preoperative brain CT or MRI examination indicating hematoma thickness, midline shift degree, and whether it was bilateral hematoma. (4) Delirium: the Intensive Care Delirium Screening Checklist (ICDSC) and the Confusion Assessment Method-Intensive Care Unit (CAM-ICU) were used to score and record the patients. The ICDSC and CAM-ICU are commonly used delirium assessment scales. The ICDSC scale includes 8 features: changes in the level of consciousness, inattention, disorientation, hallucinations and delusions, psychomotor agitation or retardation, speech and mood disturbances, sleep-wake cycle disturbances, and symptom fluctuations. Each item is scored as 0 or 1 point, with a total score of 8 points, and ≥4 points can be diagnosed as positive for delirium (14). The CAM-ICU mainly evaluates from 4 aspects, including ① the patient’s state of consciousness has acute changes or fluctuating phenomena; ② attention decline; ③ thinking disorder; ④ change in the level of clarity of consciousness. Patients who have features ① and ②, and also have features ③ and ④ are positive for delirium.

### 2.3 Statistical methods

SPSS 22.0 software was used for statistical analysis. Count data were expressed as frequency and percentage (%), and non-normally distributed measurement data were expressed as median and quartile [*M* (P25, P75)]. *T*-test or Mann–Whitney *U* rank sum test was used to compare the differences of measurement data between groups, and *χ*^2^ test was used to compare the differences of count data between groups. Lasso regression analysis was used to screen out the possible risk factors. To select the final candidate variables, we performed cross-validation and chose lambda = 1se. We then fitted a multivariate logistic regression model using these variables. The model’s performance was evaluated by the area under the receiver operating curve (AUC), sensitivity and specificity. We plotted the nomogram using the R package “regplot.” To assess the calibration of the model, we used the *C* index (bootstrap resampling 1,000 times), the calibration curve (relationship between observation probability and prediction probability), and the Hosmer–Lemeshow goodness of fit test (HL test). To assess the net clinical benefit of the model, we used decision curve analysis (DCA). All statistical analyses were completed using R language (version 3.6.3); a *p* < 0.05 was considered statistically significant.

## 3 Results

### 3.1 Patient characteristics

We retrospectively analyzed the clinical data of 208 patients with chronic subdural hematoma, of whom 109 (37.7%) developed postoperative delirium. We compared the baseline characteristics of the delirium group and the non-delirium group, and found no significant differences in gender, age, smoking history, hypertension history, cardiac history, operation duration, etc. (*p* > 0.05). However, there were significant differences in alcohol abuse history, liver dysfunction history, diabetes history, preoperative mRS score, bilateral hematoma, preoperative hematoma thickness, preoperative midline structure shift, postoperative VRS pain grade, and physical restraint (*p* < 0.05), as shown in Table 1.

**Table 1:** Demographics and clinical characteristics of patients with CSDH who underwent surgical treatment.

Variable	Total (*n* = 289)	Postoperative delirium (*n* = 109)	No postoperative delirium (*n* = 180)	*p*/*χ*^2^ value
Age, median (range), years	69 [65, 74]	69 [65, 73]	69 [64.25, 75]	0.630
Female sex, no. (%)	52 (18)	17 (15.6)	35 (19.4)	0.409
Smoking history, no. (%)	172 (59.5)	72 (66.1)	100 (55.6)	0.078
Alcoholism history, no. (%)	40 (13.8)	31 (28.4)	9 (5)	0.000
Stroke history, no. (%)	148 (51.2)	56 (51.4)	92 (51.1)	0.965
Hepatic insufficiency, no. (%)	98 (33.9)	67 (61.5)	31 (17.2)	0.000
Diabetes history, no. (%)	37 (12.8)	7 (6.4)	30 (16.7)	0.012
Hypertension history, no. (%)	66 (22.8)	25 (22.9)	41 (22.8)	0.975
Heart disease history, no. (%)	90 (31.1)	33 (30.3)	57 (31.7)	0.804
Pre-hospital mRS, median (upper and lower quartiles)	2 (2–3)	*Z* = −9.648		0.000
Bilateral hematoma, no. (%)	248 (85.8)	103 (94.5)	145 (80.6)	0.001
Preoperative hematoma thickness, median (range), mm	15.28 (7, 25)	16.28 (8, 25)	14.68 (7, 22)	0.000
Deviation distance of midline structure before surgery, median (range), mm	13.14 (1, 19)	14.16 (7, 19)	12.52 (1, 18)	0.000
Duration of surgery, median (range), minutes	90.53 (50, 163)	87.78 (56, 128)	92.19 (50, 163)	0.091
VRS postoperative pain grading, median (upper and lower quartiles)	2 (1–2.5)	*Z* = −7.414		0.000
Physical restraint, no. (%)	85 (29.4)	50 (45.9)	35 (19.4)	0.000

### 3.2 Characteristics selection and development of a nomogram

**Figure 2:**
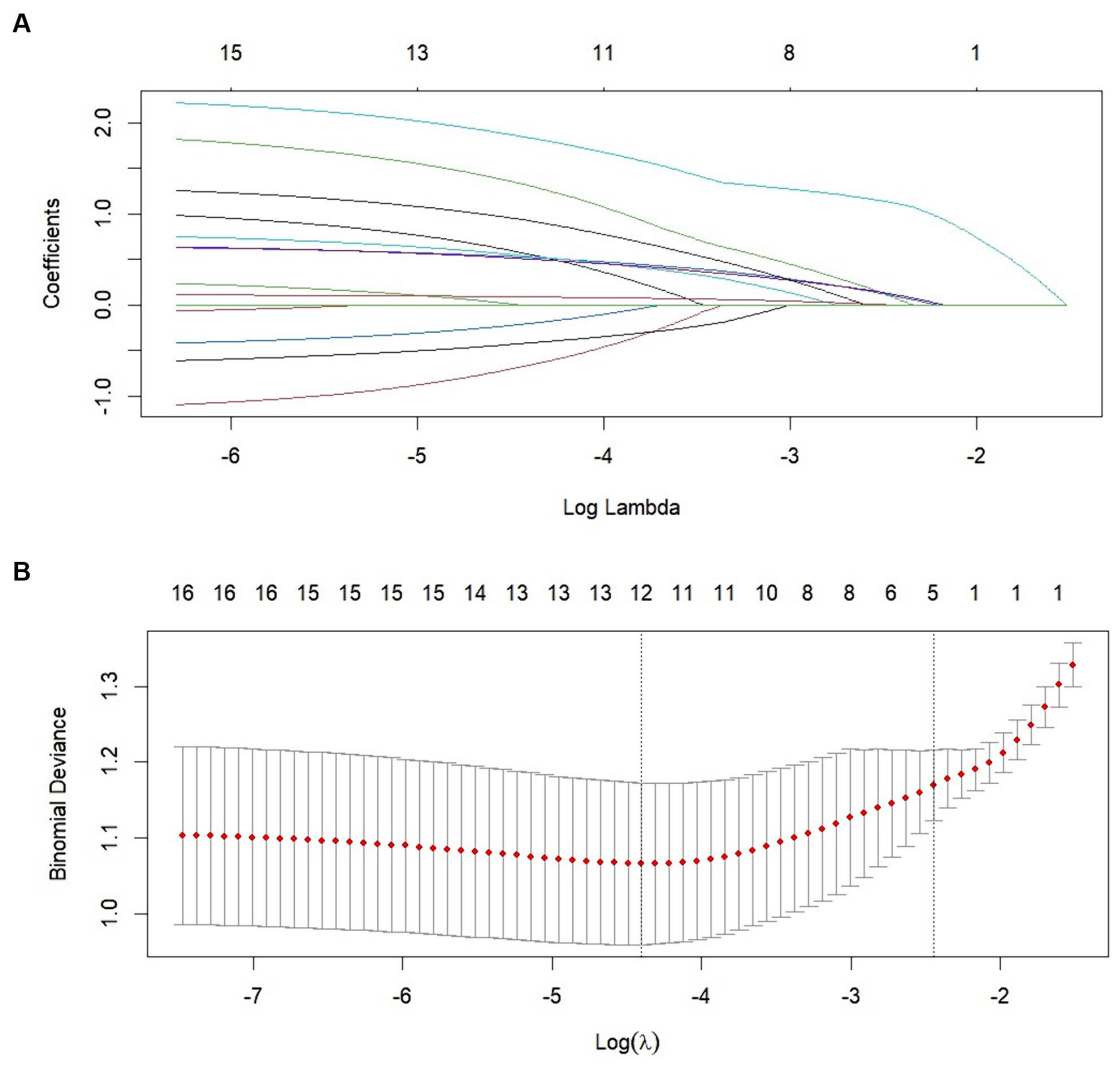
Clinical variables were selected using the lasso logistic regression model. **(A)** Tuning parameter (*λ*) selection using LASSO penalized logistic regression with 10-fold cross-validation. **(B)** LASSO coefficient profiles of the radiomic features.

**Figure 3:**
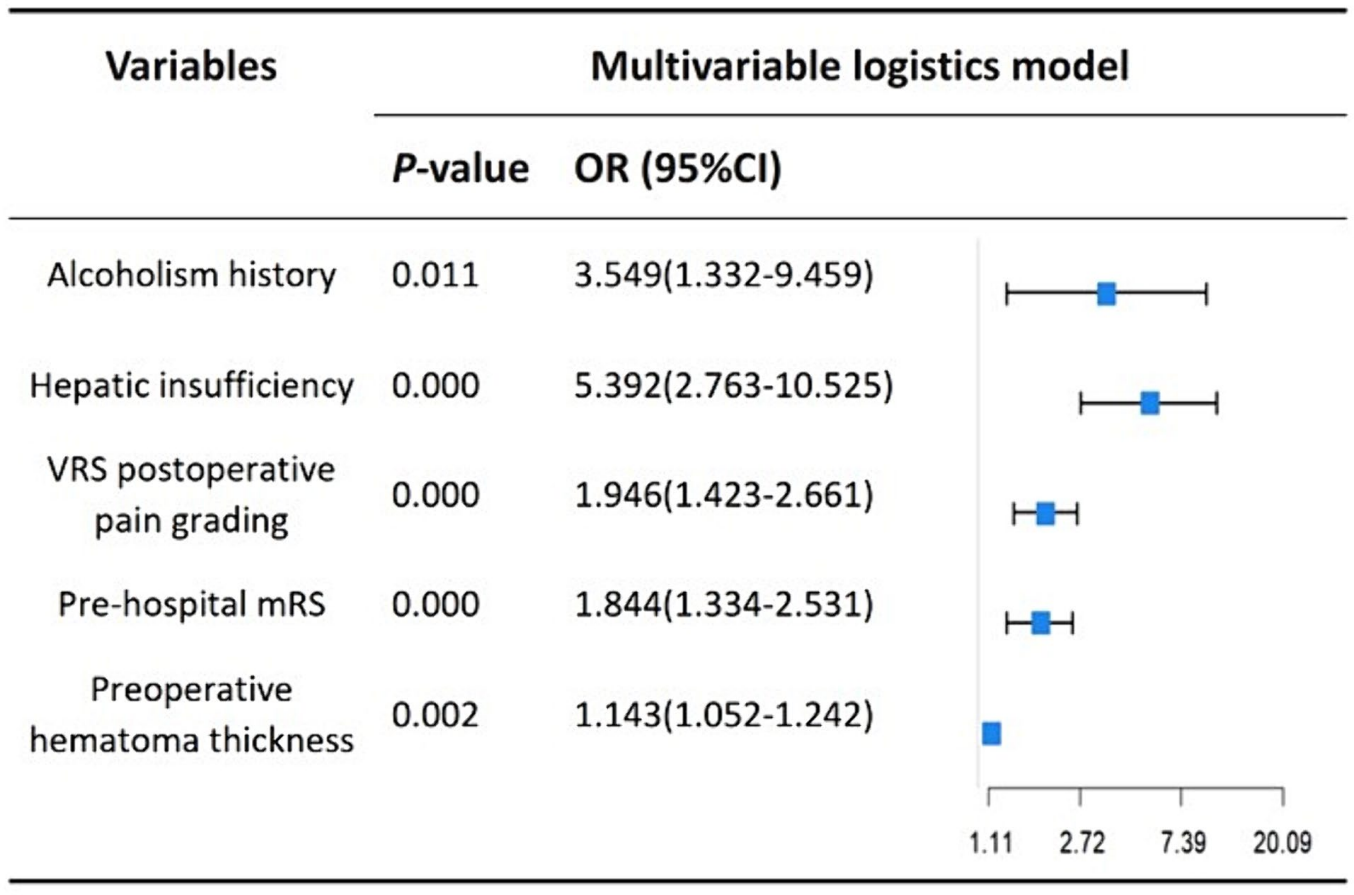
Multivariate regression model based on LASSO regression results.

**Figure 4:**
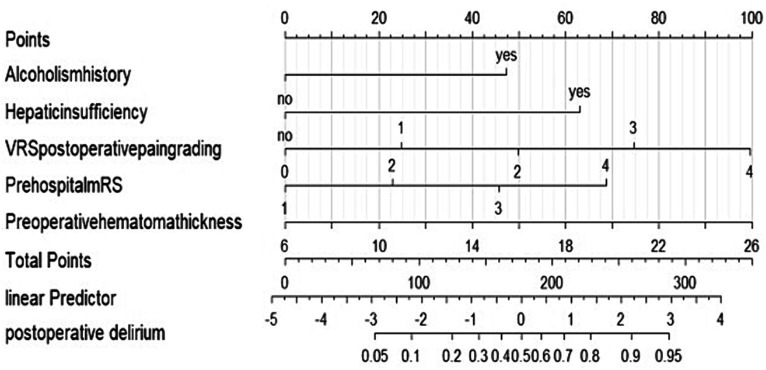
Nomogram to predict the risk of postoperative delirium after chronic subdural hematoma surgery. If a patient has a history of alcoholism, they receive 43 points and there is no history of alcoholism, they receive 0 points. If a patient has hepatic insufficiency, they receive 61 points. And if there is no hepatic insufficiency, they receive 0 points. If the postoperative pain is graded as 0, the patient receives 0 points. For a pain grade of 1, the patient is assigned 24 points. A pain grade of 2 corresponds to 49 points. If the pain is graded as 3, the patient receives 73 points. The highest pain grade, 4, results in 99 points. If a patient has a prehospital mRS grade of 1, they receive 0 points. For a prehospital mRS grade of 2, the patient is assigned 22 points. A grade of 3 corresponds to 43 points. If the prehospital mRS grade is 4, the patient receives 68 points. If the hematoma thickness is 6 mm, the patient receives 0 points. For a thickness of 10 mm, the patient is assigned 20 points. A thickness of 14 mm corresponds to 40 points. If the hematoma thickness is 18 mm, the patient receives 60 points. A thickness of 22 mm results in 80 points. The maximum thickness, 26 mm, corresponds to 100 points. The total points are calculated by summing up the points from all individual factors. These total points correlate with a linear predictor value. The linear predictor scale at the bottom of the nomogram ranges from −5 to 4. The total points correspond to a specific position on this scale. The linear predictor value is used to estimate the probability of postoperative delirium. The nomogram provides a scale for postoperative delirium probability, ranging from 0.05 to 0.95. Based on the linear predictor value, the corresponding probability can be read from this scale.

We performed lasso logistic regression on 16 variables and selected five variables based on the minimum binomial deviance criterion (ratio 5:1) (Figure 2). The final multivariate logistic regression model included the following five variables: alcoholism history (OR: 3.549; 95% CI 1.332–9.459), hepatic insufficiency (OR: 5.392; 95% CI 2.763–10.525), VRS postoperative pain grading (OR: 1.946; 95% CI 1.423–2.661), pre-hospital mRS (OR: 1.844; 95% CI 1.334–2.531), and preoperative hematoma thickness (OR: 1.143; 95% CI 1.052–1.242) (Figure 3). Based on this model, we constructed a nomogram to predict the incidence of postoperative delirium in patients with chronic subdural hematoma (Figure 4).

### 3.3 Apparent performance of the nomogram

**Figure 5:**
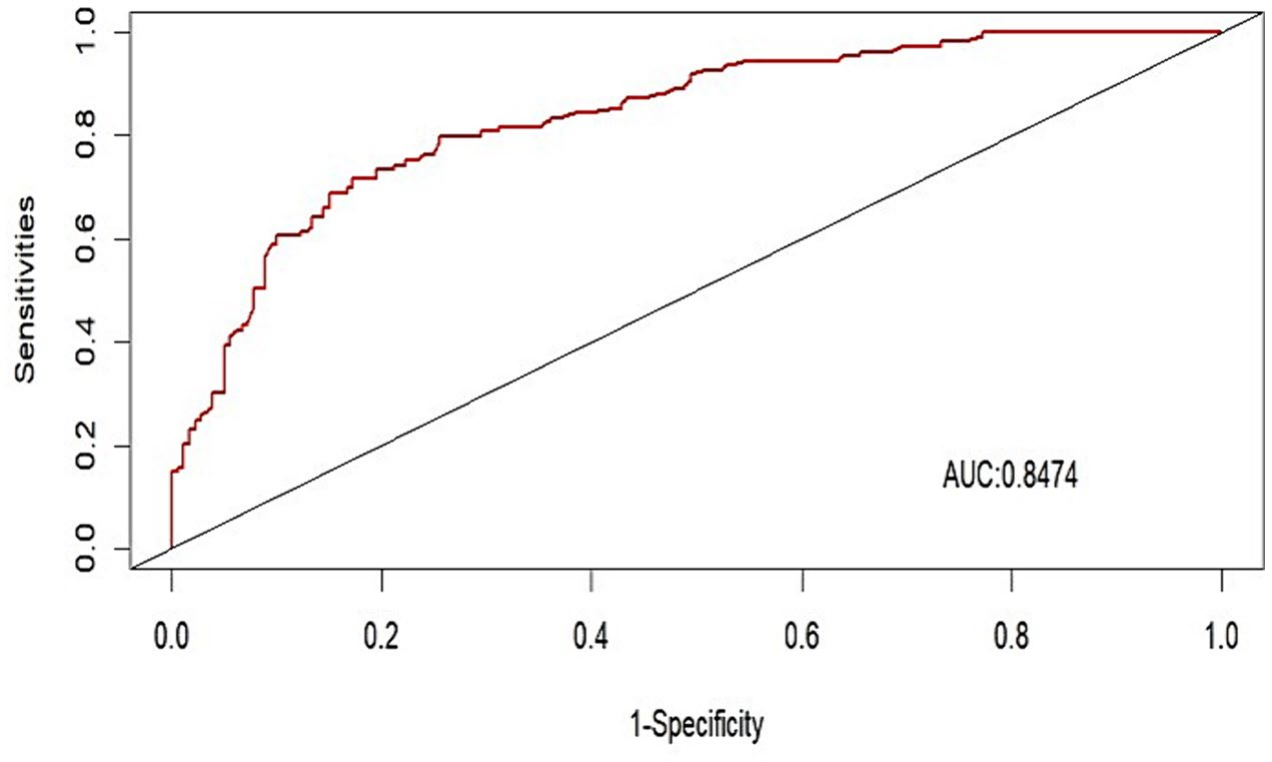
Receiver operating characteristic curve of the nomogram. AUC, area under curve.

**Figure 6:**
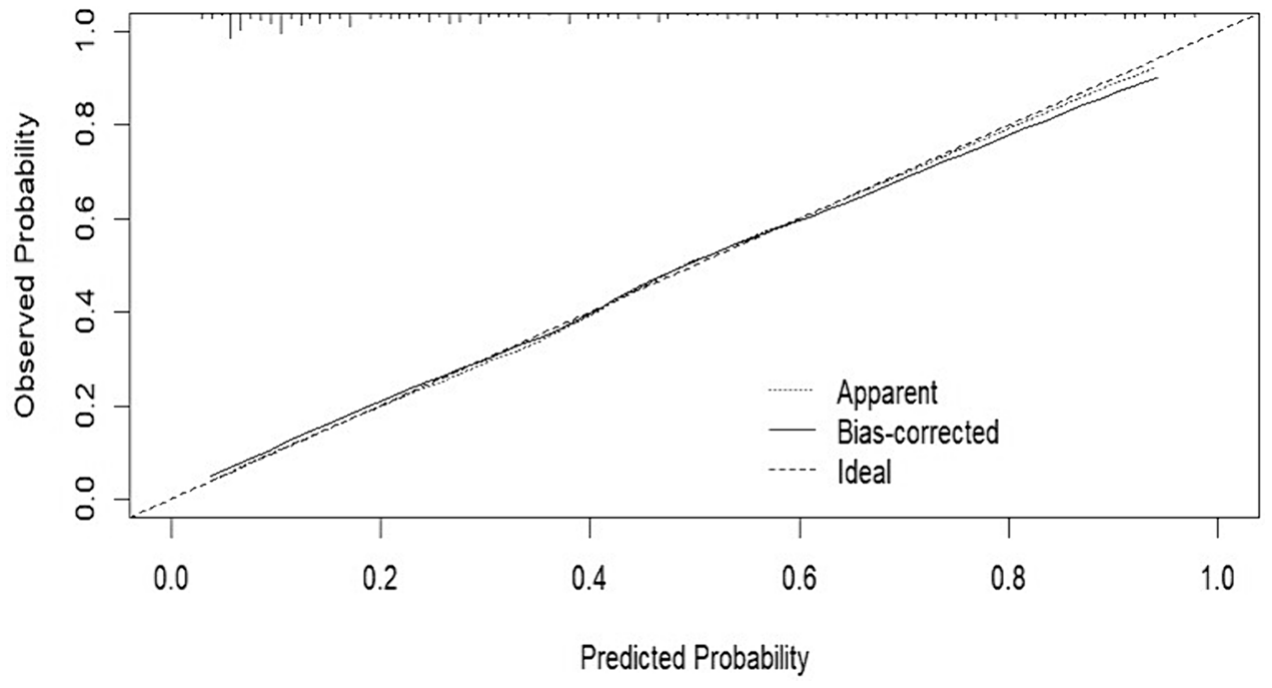
Calibration curves of the predicted nomogram. The dashed line represents the original performance, and the solid dashed line represents the performance during internal validation by bootstrapping (*B* = 1,000 repetitions). Results of the Hosmer–Lemeshow test demonstrate that the *p*-value was 0.9288.

We evaluated the performance of the nomogram by using the ROC curve and the AUC value. Our model achieved an AUC value of 0.8474 (95% CI 0.7908–0.8840), with a *C*-index of 0.8278 after 1,000 bootstrap resampling internal validations (Figure 5). The calibration curve also showed a good agreement between the predicted and observed probabilities of postoperative delirium during internal validation (Figure 6). The HL test confirmed that there was no significant difference between our predicted and observed values (*p* = 0.9288), indicating that our nomogram had good calibration ability.

### 3.4 Clinical practice

**Figure 7:**
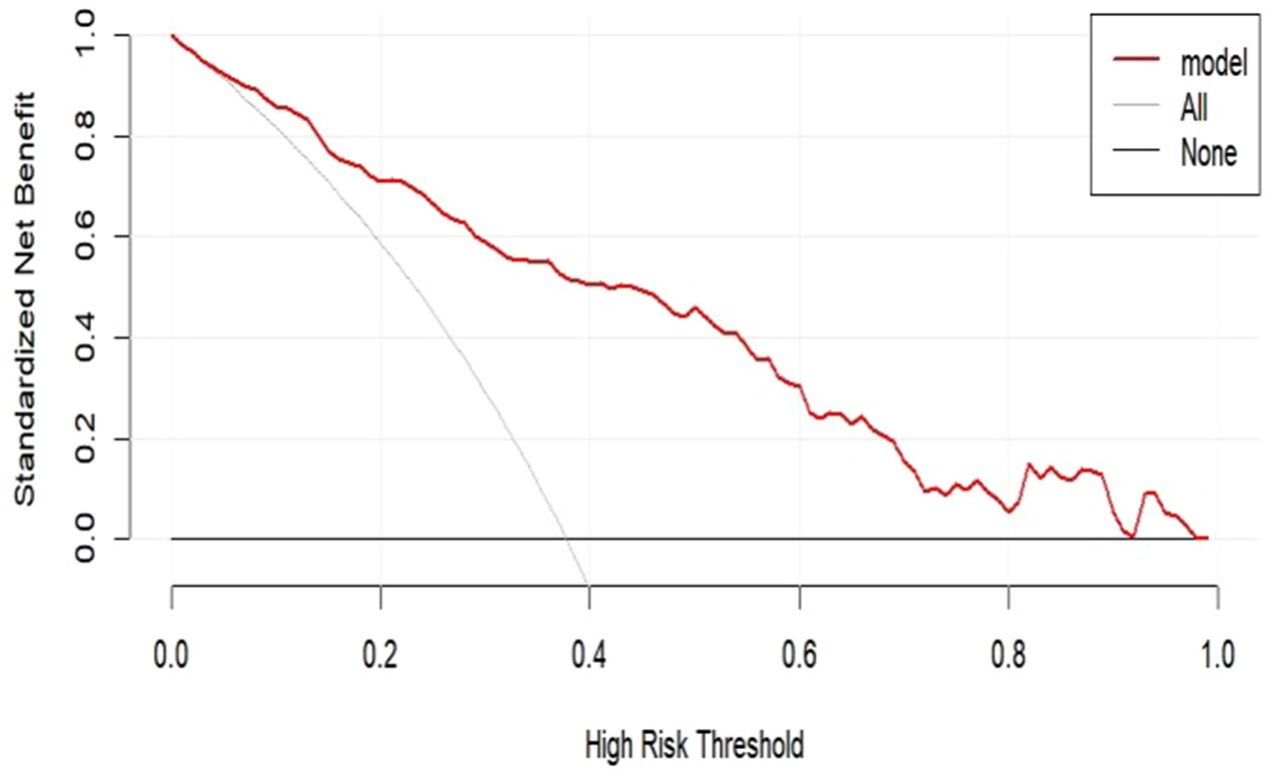
DCA for our model. The *y*-axis measures the net benefit. DCA, decision curve analysis.

**Figure 8:**
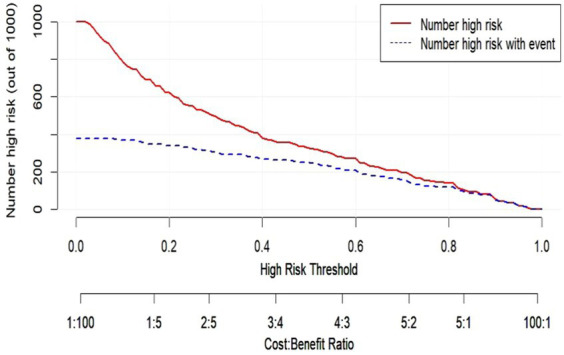
The horizontal axis is the threshold probability value and the loss-benefit ratio, the vertical axis is the incidence of postoperative delirium, the red curve (number of high risk) indicates the proportion of people who are classified as having a high risk of postoperative delirium by the model at each threshold probability, and the blue curve (number of high risk with event) indicates the proportion of people who actually develop postoperative delirium at each threshold probability.

We performed DCA of the nomogram (Figure 7). The dashed curve in the figure represents the scenario where all the patients received intervention, the solid line represents the scenario where none of the patients received intervention, and the red curve represents the clinical benefit of our model. Our results showed that our model had a superior clinical benefit with the threshold probability in the range of 0.08–0.92. Based on the DCA curve, we developed a clinical impact curve (Figure 8). We used the model to predict the risk stratification of 1,000 people, displayed the “loss: benefit” axis, displayed the confidence interval, and obtained the result shown in Figure 8. The red curve (number of high risk) indicates the number of people classified as positive (high risk) by the model at each threshold probability; the blue curve (number of high risk with outcome) is the number of true positives for each threshold probability. In summary, these results indicate that our model can accurately predict the incidence of postoperative delirium in patients with chronic subdural hematoma.

## 4 Discussion

Chronic subdural hematoma (CSDH) is a common disease that occurs mainly in elderly people. With the increase of the aging population, the incidence of CSDH is gradually rising. Currently, the preferred treatment option for CSDH is surgery. Postoperative delirium (POD) is an acute change in mental status that occurs after surgery, characterized by attention deficit and fluctuation of consciousness level, usually within 5 days after surgery (15). Delirium can prolong hospital stay and increase hospital costs, as well as increase the risk of other postoperative complications and mortality (16). This paper builds a nomogram model based on the clinical data of patients and applies it to predict the risk of postoperative delirium in patients with CSDH. A nomogram is a visual model of risk factors that has been developed in recent years. It has the characteristics of graphical indicators, which are easy to interpret by medical staff. It can more accurately predict the individual risk of outcomes, help clinical decision-making, and has high clinical value. Although many studies have discussed the related risks of postoperative delirium (15), they have not focused on CSDH as a specific disease, nor have they considered the special situation of delirium induced by the brain recovery process after CSDH surgery. We conducted a study on the relevant risk factors for CSDH, filling the gap in the stratification research of this type of patients. After internal validation of the model, we found that the prediction results of the model were consistent with the actual results. More interestingly, compared with previous studies, the model we built had better discrimination ability and net clinical benefit. In summary, we provide an easy-to-use model for this type of patients, which can identify high-risk patients early, take appropriate intervention measures early, and reduce adverse prognosis and hospital mortality.

Many scholars have studied the pathogenesis of postoperative delirium in patients with chronic subdural hematoma. Ogasawara et al. (17) found that elderly patients with chronic subdural hematoma had persistent high perfusion pressure after hematoma evacuation, which rapidly increased the capillary bed perfusion pressure, causing damage to the blood-brain barrier, leading to delirium, intracerebral hemorrhage, and even death. Rengel et al. (10) suggested that the imbalance of γ-aminobutyric acid (GABA) level, whether excessive or deficient, and the increase of 5-hydroxytryptamine concentration were related to delirium. S100β is a calcium-binding protein, which is now recognized as a biomarker of acute brain injury and increased blood-brain barrier permeability (18). The expression of S100β increased in POD patients (19), which reduced the function of the blood-brain barrier, aggravated the neuroinflammatory response, and eventually caused delirium. However, unfortunately, there is still a lack of clinical research on postoperative delirium in patients with chronic subdural hematoma. Therefore, it is necessary to develop an easy-to-use and reliable tool to inform clinical practice. In this study, we established a nomogram with five predictive factors: alcoholism history, hepatic insufficiency, VRS postoperative pain grading, pre-hospital mRS, and preoperative hematoma thickness. In Table 1, there were nine variables: alcoholism history, liver dysfunction history, diabetes history, pre-hospital mRS score, bilateral hematoma, preoperative hematoma thickness, preoperative midline structure shift, postoperative VRS pain grade, and physical restraint. After lasso regression, the final five variables entered the model were alcoholism history, liver dysfunction history, pre-hospital mRS score, preoperative hematoma thickness, and postoperative VRS pain grade. We considered diabetes history as a confounding factor, bilateral hematoma, preoperative hematoma thickness, and preoperative midline structure shift had interaction effects, which needed further study, and postoperative VRS pain grade and physical restraint had interaction effects. Lasso regression selected the most accurate preoperative hematoma thickness and postoperative VRS pain grade to enter the model. It can be seen that the variables selected by our model are very representative.

The risk factors for postoperative delirium in patients with chronic subdural hematoma include pre-existing dementia, advanced age, limb dysfunction, and rapid decompression of CSDH (17, 20, 21). In this study, we could not collect data on pre-existing dementia, limb dysfunction, and rapid decompression of CSDH due to data missing, and more studies are needed to confirm this. We did not find a close correlation between advanced age and postoperative delirium in patients with chronic subdural hematoma in this study, which may be due to the small sample size and statistical error. Although the multivariate analysis showed that male gender was one of the risk factors for postoperative mental disorder, there was no previous report on gender itself as a risk factor, and alcoholism history was a risk factor for postoperative delirium (17). Since these factors were obviously related to male gender, it might lead to the misconception that male gender was related to postoperative delirium. In this study, there was no significant correlation between male gender and postoperative delirium, but alcoholism was correlated with postoperative delirium, which confirmed this point.

Our retrospective study had several limitations. First, we did not collect data on the surgical process, which might confuse our results. The surgical process was decided by each surgeon, which should take into account the patient’s clinical condition, imaging findings, or medication. Second, in the assessment process, the preoperative evaluation might be inaccurate due to the long preoperative hospital stay. Third, the preoperative and postoperative medication could not be fully evaluated. Fourth, the sample size of this study was small, rather than a sufficient multivariate analysis. Therefore, conducting a prospective study with an adequate sample size may yield more accurate results.

## 5 Conclusion

Our study found that alcoholism history, liver dysfunction history, pre-hospital mRS score, preoperative hematoma thickness, and postoperative VRS pain grade were predictive factors for postoperative delirium in patients with chronic subdural hematoma. We established and validated a multivariate logistic regression model and a nomogram. In clinical practice, the nomogram can help doctors screen high-risk patients, optimize resource utilization, and reduce the mortality of this patient population.

## 6 Limitations

During the retrospective review of patient records, unfortunately, specific information regarding “previous dementia” was not explicitly documented in the medical records. As a result, we were unable to assess its impact as a predisposing factor. Moving forward, we will take great care to meticulously record this variable in future studies to ensure a more comprehensive analysis of its influence on the disease. Our research did not directly participate in the diagnostic process for “alcoholism,” liver dysfunction, and “previous stroke.”

## Data availability statement

The original contributions presented in the study are included in the article/supplementary material, further inquiries can be directed to the corresponding author.

## Ethics statement

Ethical review and approval was not required for the study on human participants in accordance with the local legislation and institutional requirements. Written informed consent from the patients was not required to participate in this study in accordance with the national legislation and the institutional requirements.

## Author contributions

PW: Writing – review & editing, Writing – original draft. SY: Writing – review & editing, Methodology, Conceptualization. JZhe: Writing – review & editing, Formal analysis. JL: Writing – original draft, Conceptualization. NL: Writing – original draft, Methodology. JZha: Writing – review & editing, Writing – original draft, Software, Methodology.
